# Experimental Transmission of *Leishmania infantum* by Two Major Vectors: A Comparison between a Viscerotropic and a Dermotropic Strain

**DOI:** 10.1371/journal.pntd.0001181

**Published:** 2011-06-14

**Authors:** Carla Maia, Veronika Seblova, Jovana Sadlova, Jan Votypka, Petr Volf

**Affiliations:** 1 Department of Parasitology, Faculty of Sciences, Charles University, Prague, Czech Republic; 2 Unidade de Parasitologia Médica, Centro de Malária e Doenças Tropicais, Instituto de Higiene e Medicina Tropical, Universidade Nova de Lisboa, Lisboa, Portugal; Institut Pasteur, France

## Abstract

We quantified *Leishmania infantum* parasites transmitted by natural vectors for the first time. Both *L. infantum* strains studied, dermotropic CUK3 and viscerotropic IMT373, developed well in *Phlebotomus perniciosus* and *Lutzomyia longipalpis*. They produced heavy late-stage infection and colonized the stomodeal valve, which is a prerequisite for successful transmission. Infected sand fly females, and especially those that transmit parasites, feed significantly longer on the host (1.5–1.8 times) than non-transmitting females. Quantitative PCR revealed that *P. perniciosus* harboured more CUK3 strain parasites, while in *L. longipalpis* the intensity of infection was higher for the IMT373 strain. However, in both sand fly species the parasite load transmitted was higher for the strain with dermal tropism (CUK3). All but one sand fly female infected by the IMT373 strain transmitted less than 600 promastigotes; in contrast, 29% of *L. longipalpis* and 14% of *P. perniciosus* infected with the CUK3 strain transmitted more than 1000 parasites. The parasite number transmitted by individual sand flies ranged from 4 up to 4.19×10^4^ promastigotes; thus, the maximal natural dose found was still about 250 times lower than the experimental challenge dose used in previous studies. This finding emphasizes the importance of determining the natural infective dose for the development of an accurate experimental model useful for the evaluation of new drugs and vaccines.

## Author Summary

Leishmaniasis is a disease caused by protozoan parasites which are transmitted through the bites of infected insects called sand flies. The World Health Organization has estimated that leishmaniases cause 1.6 million new cases annually, of which an estimated 1.1 million are cutaneous or mucocutaneous, and 500,000 are visceral, the most severe form of the disease and fatal if left untreated. The development of a more natural model is crucial for the evaluation of new drugs or vaccine candidates against leishmaniases. The main aim of this study was to quantify the number of *Leishmania infantum* parasites transmitted by a single sand fly female into the skin of a vertebrate host (mouse). Two *L. infantum* strains, viscerotropic IMT373 and dermotropic CUK3, were compared in two natural sand fly vectors: *Phlebotomus perniciosus* and *Lutzomyia longipalpis*. We found that the parasite number transmitted by individual sand flies ranged from 4 up to 4.19×10^4^. The maximal natural infective dose found in our experiments was about 250 times lower than the experimental challenge dose used in most previous studies.

## Introduction

*Leishmania* are intracellular protozoan parasites that establish infection in mammalian hosts following transmission through the bite of an infected phlebotomine sand fly. Visceral leishmaniasis, caused by *Leishmania donovani* in the Old World and *L. infantum* in both the Old and New World, invariably leads to death if left untreated [1]. Despite the fact that parasites from the *L. donovani* complex are mainly associated with disseminated infection of the spleen and liver, it has been shown that *L. infantum* can also cause cutaneous lesions [2]–[5]. A novel focus of cutaneous leishmaniasis caused by *L. infantum* was recently described in the Cukurova region in Turkey [6].

During the natural transmission of *Leishmania* into the dermis, sand flies deposit pharmacologically active saliva [7] and egest parasite-released glycoconjugates, the promastigote secretory gel [8]. Both substances modulate the immune response of the bitten host and enhance the severity of infection (reviewed by [9]).

The ideal leishmaniasis model to test therapeutics and immunoprophylaxis candidates should reproduce the biological and immunological aspects of natural infection and disease. Different approaches regarding the parasite number and route of inoculation have been tested in order to develop an accurate experimental model for the *L. donovani* complex, most of them using subcutaneous, intraperitoneal or intravenous injections of millions of axenic promastigotes or amastigotes [10]–[11]. Although in some studies up to 10^7^ parasites have been co-inoculated into the dermis with small amounts of sand fly saliva, is not clear how well these experiments mimic natural transmission [12]–[13].

The number of *L. infantum* parasites inoculated by infected vectors during natural transmission was not previously known, even though a determination of the natural infective dose is crucial for the development of an accurate experimental model to evaluate new drugs and vaccine candidates. In the *L. major - P. duboscqi* model, it was demonstrated that the number of promastigotes inoculated by individual sand flies ranged between 10 and 1×10^5^ *Leishmania* [14]. The average number of *L. infantum* parasites egested was recently reported [15], but the technique used (feeding the pool of infected *L. longipalpis* through chick skin membrane on culture medium) did not allow an evaluation of the variation in numbers delivered by individual sand flies. Thus, the main aims of this work were to determine the transmission rate and the number of promastigotes inoculated into the skin of mice by individual sand fly females. *Phlebotomus perniciosus* and *Lutzomyia longipalpis,* two main vectors of *L. infantum* in the Mediterranean basin and in the New World, respectively [16], were experimentally infected by *L. infantum* dermotropic and viscerotropic parasites.

## Results

The following results summarize the data obtained in 15 and 10 independent experiments with both vectors and *L. infantum* strain combinations: 9 with *P. perniciosus*-IMT373, 6 with *P. perniciosus*-CUK3, 6 with *L. longipalpis*-IMT373 and 4 with *L. longipalpis*-CUK3.

### Experimental infections of sand flies: comparison of IMT373 and CUK3 strains

The *L. infantum* strains studied developed well in both *P. perniciosus* and *L. longipalpis*, producing heavy late-stage infection and colonizing the stomodeal valve of the vectors, which is a prerequisite for successful transmission. For both *L. infantum* strains, the average parasite load in the sand fly midgut is summarized in Table 1. Quantitative PCR revealed that in *P. perniciosus* the intensity of infection was higher for the CUK3 strain (*p* = 0.01) while *L. longipalpis* harboured more IMT373 parasites (*p*<0.001). However, in both sand fly species the number of parasites transmitted was higher for the dermotropic strain CUK3 (*p*<0.001); see below.

**Table 1:** Pre-feeding and transmitted parasite load for *L. infantum* strains by both sand fly species.

	*L. infantum* IMT373		*L. infantum* CUK3	
	*P. perniciosus*	*L. longipalpis*	*P. perniciosus*	*L. longipalpis*
Parasite load in sand fly midgut* (mean/median)	65 768/52 506	154 433/79 691	499 500/114 963	79 888/27 854
Transmitted parasites (mean/median)	88/28	104/24	2 350/29	1127/13
Percentage of transmission** (mean/median)	0.47%/0.07%	0.19%/0.04%	0.5%/0.02%	2.3%/0.03%

*Parasite load was calculated as a sum of midgut parasites plus those transmitted by bite.

**Percentage of parasite load transmitted by bite.

### Transmission of the dermotropic strain CUK3

**Figure 1:**
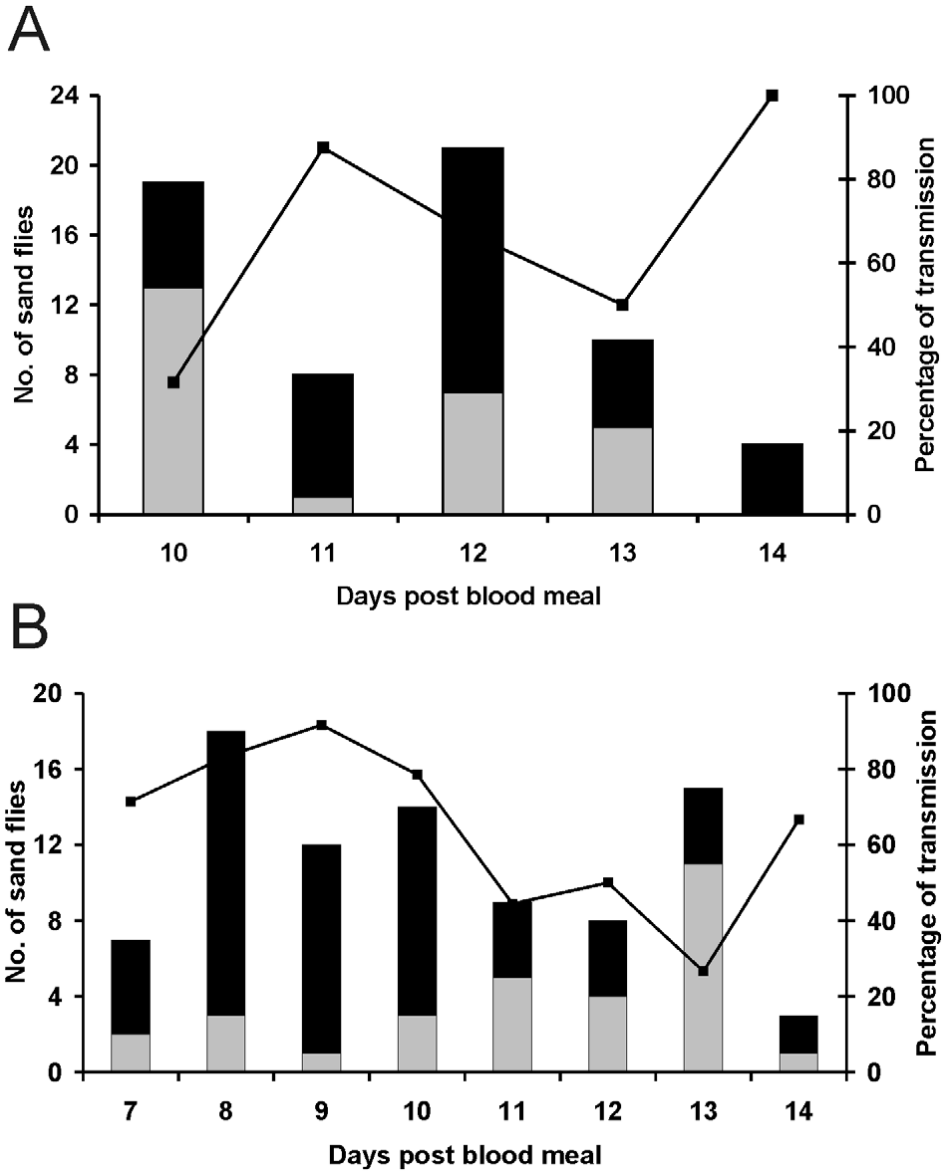
Transmission of *Leishmania infantum* CUK3. Percentage transmission of CUK3 strain by experimentally infected *Phlebotomus perniciosus* (A) and *Lutzomyia longipalpis* (B). Black bars, infected females that transmitted by bite; grey bars, infected females that did not transmit; line, percentage of females that transmitted parasites.

Out of 88 *P. perniciosus*, females that bit mice, 62 (70.5%) were infected with CUK3; of these, 36 (58%) delivered parasites into the skin of the mice on days 10–14 post infective blood meal (Fig. 1a). Out of 114 biting *L. longipalpis* females, 86 (75.5%) were infected and 56 (65% of those infected) inoculated parasites into the mice on days 7–14 post infective blood meal (Fig. 1b).

Despite the fact that the intensity of infection was significantly higher in *P. perniciosus* (*p*<0.01), the percentage of transmission and number of inoculated parasites was comparable for both vectors. The parasite load delivered by *P. perniciosus* and *L. longipalpis* in the skin of mice ranged between 16 and 4.19×10^4^ and between 4 and 1.11×10^4^, respectively. The average number of CUK3 parasites inoculated into the skin of mice and the percentages of transmission are summarized in Table 1.

In *L. longipalpis*, the feeding time was positively correlated with the number of CUK3 parasites delivered into host skin (*p*<0.05), while in *P. perniciosus* females no such correlation was observed. On the other hand, there was a significant correlation between the pre-feeding load inside both sand fly species' midguts and the number of parasites transmitted (*p* = 0.0178 for *L. longipalpis* and *p*<0.001 for *P. perniciosus*).

### Transmission of the viscerotropic strain IMT373

**Figure 2:**
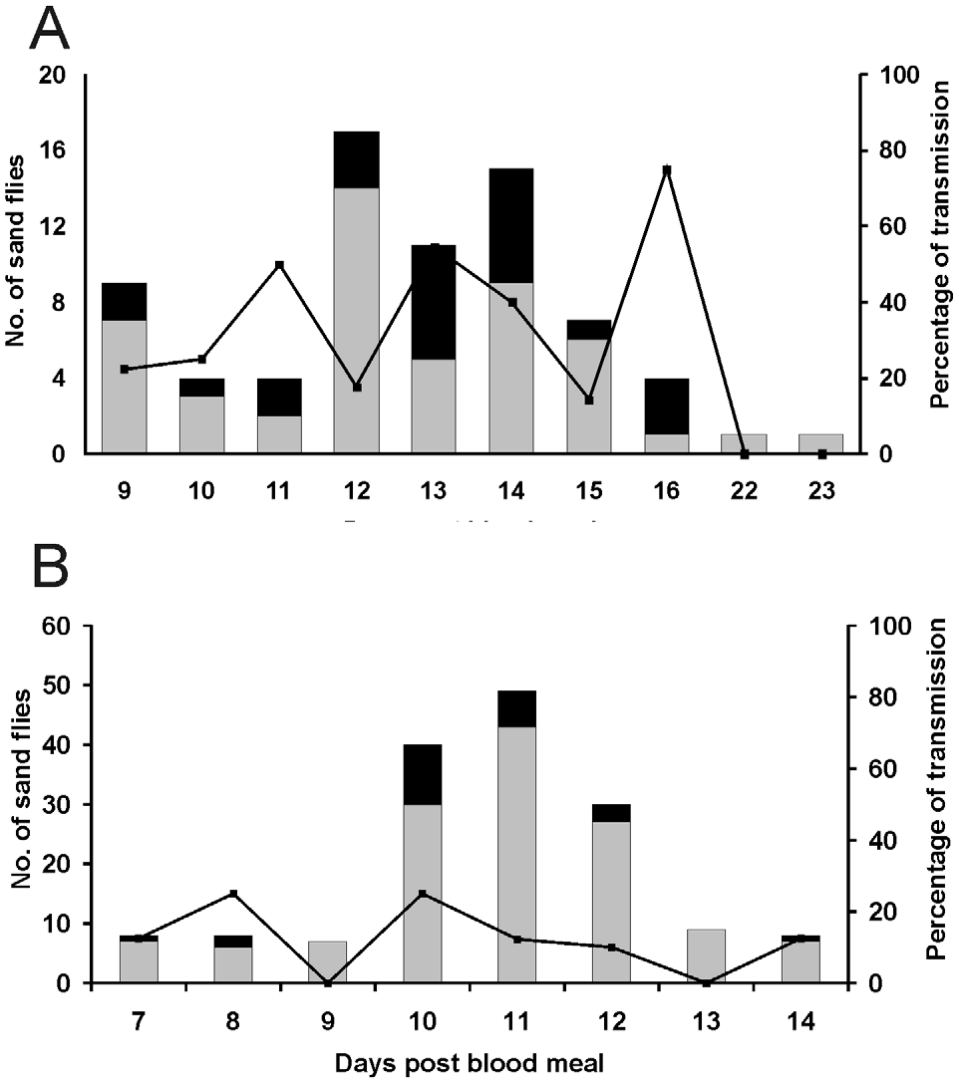
Transmission of *Leishmania infantum* IMT373. Percentage transmission of IMT373 strain by experimentally infected *Phlebotomus perniciosus* (A) and *Lutzomyia longipalpis* (B). Black bars, infected females that transmitted by bite; grey bars, infected females that did not transmit; line, percentage of females that transmitted parasites.

Out of 101 *P. pernicious* females that bit mice, 73 (72%) were infected with IMT373, and of these 24 (33%) transmitted parasites into the mice's skin. *Leishmania* transmission occurred between days 9 and 16 post infective bloodmeal (Fig. 2a). From 190 biting *L. longipalpis* females, 159 (84%) were infected and 23 (14.5% on infected ones) inoculated parasites into the mice between days 7 and 14 post blood meal (Fig. 2b).

In contrast to above, the intensity of infection was significantly higher in *L. longipalpis* (*p*<0.001), but the transmission rate (i.e. percentage of transmitting females) and the number of parasites transmitted were significantly higher in *P. perniciosus* (*p*<0.01).

The number of parasites transmitted by *P. perniciosus* and *L. longipalpis* ranged from 8 to 513 and between 7 and 1240 promastigotes, respectively. The median number of IMT373 transmitted is summarized in Table 1.

For both sand fly species, there was no correlation between feeding time and the number of IMT373 parasites in each female (*p* = 0.1594), or between the time to take a blood meal and the number of parasites transmitted (*p* = 0.6666). Moreover, no correlation was observed between the pre-feeding load in each sand fly species and the number of *Leishmania* delivered (*p* = 0.1340 for *P. perniciosus*; *p* = 0.6473 for *L. longipalpis*).

### Biting sites and feeding time of transmitting females

For all *Leishmania*-sand fly combinations, ears were the preferential biting place for sand flies transmitting the parasites, followed by the paws and tail. A few specimens that fed in the nose and eyes were also able to transmit parasites.

Table 2 summarizes the feeding times for both sand fly species: *L. longipalpis* transmitting IMT373 completed their bloodmeals in times ranging from 2 to 27 minutes, while those transmitting CUK3 parasites needed between 3 to 55 minutes. The maximum and minimum feeding times for *P. perniciosus* transmitting CUK3 and IMT373 parasites ranged between 4–33 and 1–32 minutes, respectively. Infected sand flies transmitting CUK3 needed more time to feed than those that were infected but non-transmitting, while no differences in feeding time were observed between transmitting and non-transmitting females with IMT373 parasites.

**Table 2:** Average feeding time of infected and noninfected *P. perniciosus* and *L. longipalpis*.

	*L. infantum* IMT373		*L. infantum* CUK3	
	*P. perniciosus*	*L. longipalpis*	*P. perniciosus*	*L. longipalpis*
Non-infected	10	11	8	10
Infected but without transmission	11	10	9	12
Infected and with transmission	12	10	12	18

Average time necessary for non-infected and infected sand flies to feed on mice is given in minutes.

## Discussion

For the first time, we have quantified the number of parasites belonging to *L. infantum* dermotropic and viscerotropic strains transmitted to the dermis of experimental mice by individual sand fly females. The only previous attempt to calculate the number of transmitted *L. infantum* parasites was performed just recently [15], with the average number of promastigotes inoculated by 63 *L. longipalpis* into culture medium through a chicken membrane skin being 457 parasites, with 95% (431 promastigotes) of these corresponding to metacyclic parasites. However, these results do not allow us to take into consideration the individual variability of parasite transmission by a single specimen. The wide range of parasites inoculated per individual sand fly in our study (from 4 up to 4.19×10^4^ promastigotes) is in accordance to data previously obtained with other *Leishmania*-vector combinations [14], [17], although the approach using microcapillaries as artificial feeding systems [17] could have interfered with the normal sand fly feeding behaviour.

In our study, *Phlebotomus perniciosus* harboured more *L. infantum* dermotropic parasites of the CUK3 strain, while in *L. longipalpis* the intensity of infection was higher for the viscerotropic strain IMT373. However, in both sand fly species the parasite load transmitted was higher for the strain with dermal tropism. All but one sand fly female infected by IMT373 strain transmitted less than 600 promastigotes, the exception being a *L. longipalpis* female that inoculated 1240 parasites. On the other hand, 29% of *L. longipalpis* and 14% of *P. perniciosus* infected with the CUK3 strain transmitted more than 1000 parasites.

The majority of transmitting females inoculated less than 600 parasites. As most of these females were fully engorged by blood we may expect that their feeding pumps (the cibarial and pharyngeal pumps) and stomodeal valve were functioning normally. On the other hand, in those transmitting more than 1000 parasites there was a significant correlation between the pre-feeding load and the number of parasites transmitted. We suggest that these females with high dose deliveries regurgitated parasites because of impaired stomodeal valve function [18]. This would be consistent with previous studies [19], [20] which have demonstrated an opened stomodeal valve due to the physical presence of a parasite plug and damage of the chitin layer of the valve by *Leishmania* chitinase.

Infected sand fly females, and especially those that transmit parasites, feed longer on hosts than non-transmitting ones do. *Lutzomyia longipalpis* females transmitting dermotropic CUK3 strain parasites took an average of 1.5 times longer to complete a bloodmeal compared to specimens infected but not transmitting, and 1.8 times longer than uninfected females. Similarly, *P. perniciosus* infected by CUK3 and IMT373 take 1.5 and 1.2 times more time for a blood meal. Most of the infected sand flies exposed to anaesthetized mice did not demonstrate increased probing, but rather remained feeding for longer periods until either they were fully or partially engorged. This is in agreement with data previously published on the *L. longipalpis-L. mexicana* combination [21].

Although only one dermotropic and one viscerotropic *L. infantum* strains were evaluated, the significant variation in inoculum size between them allow us to hypothetise that the infectious dose delivered by vector sand flies may be an inherent character of each *Leishmania* strain. Moreover, the infectious dose might be a determining factor in the outcome of *Leishmania* infection. Local cutaneous lesions might result from a high-dose inoculum of dermotropic *Leishmania* resulting in a strong local immune response, whereas dissemination to internal organs might be the result of infected sand flies delivering a low number of parasites below the threshold required to produce/develop a localized and restraining immune response. This hypothesis corresponds with the data of Kimblin et al. [14] on the *L. major-P. duboscqi* combination. These authors evaluated the impact of inoculum size on infection outcome by comparing *L. major* infections with high (5×10^3^) and low (1×10^2^) dose intradermally inoculated by needle in the ears of C57BL/6 mice, and observed the rapid development of large lesions in the ears of mice receiving the high-dose inoculum. In contrast, the low dose resulted in only minor pathology but a higher parasite titre during the chronic phase [14]. Nevertheless, it will be necessary to evaluate more *L. infantum* strains with visceral and cutaneous tropism in order to determine if differences detected in our study were due to individual stock characteristics or if they are associated with parasite tropism in vertebrate hosts.

In conclusion, we have demonstrated that individual sand flies transmit *Leishmania* parasites in a wide dose range. However, the maximal natural dose found was still about 250 times lower than the challenge dose used for the *L. donovani* complex in most previous experimental works. This finding emphasizes the importance of determining the natural infective dose for the development of an accurate experimental model, which is crucial for the evaluation of new drugs and vaccine candidates against leishmaniasis.

## Materials and Methods

### Parasite strains

The viscerotropic *Leishmania infantum* strain IMT373 MON-1 (MCAN/PT/2005/IMT373) and the dermotropic *L. infantum* strain CUK3 (ITOB/TR/2005/CUK3) were used in this study. CUK3 was isolated from *Phlebotomus tobbi* from a Cukurova focus of cutaneous leishmaniasis [6] while IMT373 was isolated from a dog with leishmaniasis and passaged through mice in order to keep its virulence [13], [22]. Promastigotes (with less than 12 *in vitro* passages since isolation) were cultured at +26°C in M199 medium (Sigma, USA) containing 10% heat-inactivated foetal calf serum (Gibco, USA), 50 mg/ml mikacin solution (Bristol-Myers Squibb, Czech Republic) and 1% sterile urine.

### Sand flies and experimental infections

*Lutzomyia longipalpis* (originating from Jacobina, Brazil) and *Phlebotomus perniciosus* (originating from Murcia, Spain) colonies were maintained in an insectary under standard conditions as described by Volf and Volfova [23]. Five to six-day old female flies (200 *P. perniciosus* and 150 *L. longipalpis* females per experiment, respectively) were fed on heat inactivated rabbit blood containing promastigotes (10^7^ parasites per ml of blood) through a chicken-skin membrane. Blood-engorged females were separated immediately and maintained on a 50% sucrose diet in >70% relative humidity at +26°C.

One group of females was dissected to study the development and localization of infection in the sand fly midgut two and ten days post blood meal, i.e., during early and late stage infection, respectively. Individual midguts were placed into a drop of saline buffer, and parasite numbers were estimated under a light microscope at 200X and 400X magnifications by an experienced worker. Parasite loads were graded as previously described [24] into four categories: negative, 1–100, 100–1000, and >1000 parasites per gut. A second group of females from the same batch was used for transmission experiments and parasite quantification by Real-time PCR (see below). Nine and six independent experiments were performed with *P. perniciosus*-IMT373 and *P. perniciosus*-CUK3 combinations, respectively, while six and four artificial infections were done with *L. longipalpis*-IMT373 and *L. longipalpis*-CUK3 combinations.

### Mice

One hundred and eight BALB/c mice (41 for *P. perniciosus*-IMT373, 28 mice for *L. longipalpis*-IMT373, 23 for *P. perniciosus*–CUK3 and 16 for *L. longipalpis*-CUK3 combinations) older than 8 weeks of age were purchased from AnLab (Czech Republic) and housed at Charles University, Prague, under stable climatic and dietary conditions. Experiments were approved by the institutional Ethical Committee and performed in accordance with national legislation for the care and use of animals for research purposes. Mice were anaesthetized intraperitoneally with ketamine (150 mg/kg) and xylazine (15 mg/kg).

### Transmission by bite and sample collection

Sand fly females were allowed to feed on whole body of anesthetised mice in a rectangular cage (20×20 cm) for about one hour at various days post infective blood meal (7–14 days for *L. longipalpis* and 9–23 days for *P. perniciosus*). Each mouse was placed individually into a cage together with about 50 *P. perniciosus* or 10 *L. longipalpis* females (the difference was due to the fact that *L. longipalpis* were more aggressive and had higher feeding rate). Two people followed each experiment; one recorded biting place and feeding time while the second ensured that each sand fly female probed in different place and then collected engorged flies by an aspirator immediately after terminating their blood meal; the site of bite and time of feeding were recorded for each female. After exposure, mice were sacrificed, biting place was inspected under a stereoscope and excised. Both samples (skin biopsies and corresponding fed sand flies) were stored at −20°C until DNA extraction.

### Real-time PCR (qPCR)

Extraction of total DNA from each bite site and the corresponding sand fly were performed using a DNA tissue isolation kit (Roche Diagnostics, Germany) according to the manufacturer's instructions. DNA was eluted in 100 µl and stored at −20°C. qPCR for detection and quantification of *Leishmania* sp. was performed in a Rotor-Gene 2000 from Corbett Research (St. Neots, UK) using the SYBR Green detection method (iQ SYBR Green Supermix, Bio-Rad, Hercules, CA). For adequate sensitivity, kinetoplast DNA was chosen as the molecular target, with primers as previously described [25] (forward primer 5′-CTTTTCTGGTCCTCCGGGTAGG-3′ and reverse primer 5′-CCACCCGGCCCTATTTTACACCAA- 3′). Two microliters of eluted DNA was used per individual reaction. PCR amplifications were performed in duplicate wells using the conditions described previously [26]. Briefly, 3 min at 95°C followed by 45 cycles of: 10 s at 95°C, 10 s at 56°C, and 10 s at 72°C. Reaction specificities were checked for all samples by melting analysis. Quantitative results were expressed by interpolation with a standard curve included in each PCR run. Mass cultures of *L. infantum* promastigotes were used to construct a series of 10-fold dilutions ranging from 10^5^ to 1 parasite per PCR reaction. Diluted parasites were co-processed with mouse tissue or sand fly females for DNA extraction. DNA from uninfected sand flies and mice were used as a negative control.

For sand fly females transmitting promastigotes into mouse skin, the pre-feeding midgut load was calculated as the sum of parasites in the midgut after feeding and the number of parasites transmitted.

### Statistical analysis

Statistical analysis was performed using the software STATISTICA. For each *L. infantum* strain, a nonparametric Kruskal-Wallis test was used to compare: (i) the intensity of infection in *P. perniciosus* and *L. longipalpis*, and (ii) the number of parasites transmitted by each sand fly species into mice's skin. Correlations between feeding time and the number of parasites (i) in each sand fly female and (ii) inoculated into the skin, as well as the correlation between pre-feeding load and the number of parasites transmitted, were determined by simple linear regression analysis. Differences were considered statistically significant for *p* values <0.05.
